# Spectral Grouping of Electrically Encoded Sound Predicts Speech-in-Noise Performance in Cochlear Implantees

**DOI:** 10.1007/s10162-023-00918-x

**Published:** 2023-12-07

**Authors:** Inyong Choi, Phillip E. Gander, Joel I. Berger, Jihwan Woo, Matthew H. Choy, Jean Hong, Sarah Colby, Bob McMurray, Timothy D. Griffiths

**Affiliations:** 1 Department of Communication Sciences and Disorders, University of Iowa, 250 Hawkins Dr., Iowa City, IA 52242 USA; 2 Department of Otolaryngology–Head and Neck Surgery, University of Iowa Hospitals and Clinics, Iowa City, IA 52242 USA; 3 Department of Neurosurgery, University of Iowa Hospitals and Clinics, Iowa City, IA 52242 USA; 4 Department of Radiology, University of Iowa Hospitals and Clinics, Iowa City, IA 52242 USA; 5 Department of Biomedical Engineering, University of Ulsan, Ulsan, Republic of Korea; 6 Biosciences Institute, Newcastle University, Newcastle upon Tyne, NE1 7RU UK; 7 Department of Psychological and Brain Sciences, University of Iowa, Iowa City, IA 52242 USA

**Keywords:** Cochlear implants, Auditory grouping, Speech-in-noise

## Abstract

**Objectives:**

Cochlear implant (CI) users exhibit large variability in understanding speech in noise. Past work in CI users found that spectral and temporal resolution correlates with speech-in-noise ability, but a large portion of variance remains unexplained. Recent work on normal-hearing listeners showed that the ability to group temporally and spectrally coherent tones in a complex auditory scene predicts speech-in-noise ability independently of the audiogram, highlighting a central mechanism for auditory scene analysis that contributes to speech-in-noise. The current study examined whether the auditory grouping ability also contributes to speech-in-noise understanding in CI users.

**Design:**

Forty-seven post-lingually deafened CI users were tested with psychophysical measures of spectral and temporal resolution, a stochastic figure-ground task that depends on the detection of a figure by grouping multiple fixed frequency elements against a random background, and a sentence-in-noise measure. Multiple linear regression was used to predict sentence-in-noise performance from the other tasks.

**Results:**

No co-linearity was found between any predictor variables. All three predictors (spectral and temporal resolution plus the figure-ground task) exhibited significant contribution in the multiple linear regression model, indicating that the auditory grouping ability in a complex auditory scene explains a further proportion of variance in CI users’ speech-in-noise performance that was not explained by spectral and temporal resolution.

**Conclusion:**

Measures of cross-frequency grouping reflect an auditory cognitive mechanism that determines speech-in-noise understanding independently of cochlear function. Such measures are easily implemented clinically as predictors of CI success and suggest potential strategies for rehabilitation based on training with non-speech stimuli.

**Supplementary Information:**

The online version contains supplementary material available at 10.1007/s10162-023-00918-x.

## Introduction

Although cochlear implants (CIs) have been a singularly successful intervention for patients with severe sensorineural hearing loss, variability in speech perception outcomes among CI users remains a pervasive issue [1]. Much of this variability derives from peripheral factors related to the electrode-neuron interface such as the electrode placement [2], inflammatory intracochlear responses to electrodes [3], and the degree of neural trauma and health [4] that may affect current spread. Indeed, reducing the current spread through programming changes improves spectral resolution [5–8], while it is evident that spectral resolution is correlated with speech perception performance in cochlear implant users [9–17]. The electrode configuration and encoding strategy also affect temporal resolution (as reflected in gap detection performance: [18]) as well as changes in the frequency mapping between the implant and the auditory nerve [19]. Nonetheless, even established CI users with similar audiometric profiles differ in performance, particularly for listening to speech in noise [20, 21]. This suggests that additional variation in perceptual and cognitive processes may account for some differences in speech perception. However, the neural and computational mechanisms that underlie these central processes are poorly understood.

The ability to unmask speech from noise is an example of auditory scene analysis (ASA) [22]. This entails multiple sensory and cognitive operations including (1) sensory encoding of the acoustic signal, (2) grouping and separation of acoustic features to form auditory objects (Darwin, 1997), and (3) across-object competition. Individual differences in speech-in-noise understanding may originate from each of these. In normal-hearing (NH) listeners, there is evidence that speech-in-noise success is related to individual differences in some ASA subskills, including encoding of suprathreshold dynamics [23] and auditory grouping [24, 25].

**Fig. 1:**
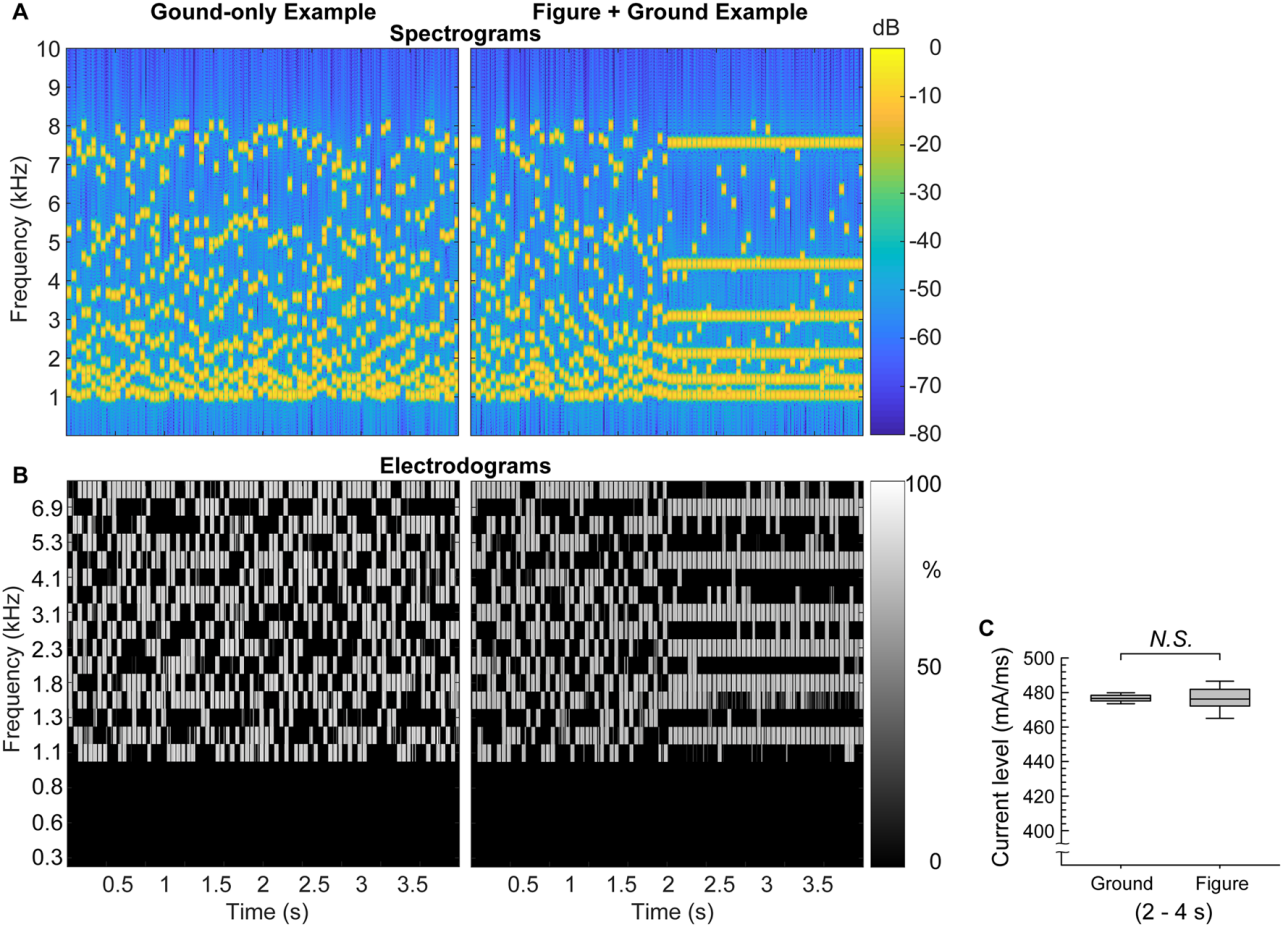
**A** Example stimulus spectrograms for the two trial types of the figure-ground task. **B** Electrodograms of the two example stimuli. **C** Comparison of integrated current levels between Ground-only and Figure+Ground stimuli in the 2–4 s period where the emergence of a “figure” is expected. N.S. indicates no significant difference found from a Mann-Whitney Rank Sum test (*p* = 0.94)

In CI users, a large portion of previous work investigating variability in speech-in-noise outcomes has focused on the first process above, the quality of the sensory encoding carried out by the peripheral auditory system in conjunction with the CI [14, 26]. Auditory stream segregation has also been studied in the CI population. Earlier studies reported that spectral separation (i.e. electrode position) is an important cue for CI users’ stream segregation of repetitive A-B-A alternating tone sequences [27, 28], while a later study found that CI users could segregate streams with the temporal cue (i.e., pulse rate) alone [29]. For the task of segregating a melody from randomly interleaved tones, CI users relied more on intensity and temporal envelope information than on fundamental frequency and spectral envelope information, although all four of the aforementioned cues contributed to the performance significantly [30]. Paredes-Gallardo et al. also reported that CI users can use both place (i.e., the electrode position) and temporal (i.e., the pulse rate) information to separate concurrent tone sequences [31, 32]. The degree of endogenous attention that facilitates the segregation also showed a relationship with speech-in-noise perception [33, 34]. However, most ASA studies in CI users utilized relatively simple tone stimuli (e.g., [35]), from which it is difficult to draw conclusions about (1) how CI users perform auditory grouping of complex auditory scenes, and (2) how such an auditory grouping ability contributes to speech-in-noise perception in CI users. Given the dramatic degradation of the auditory input in CI users, does variability in higher-order auditory-cognitive processes matter as much? To address this question, the present study tests the contribution of mechanisms for grouping together elements of an auditory object that have different frequencies to speech-in-noise understanding in CI users. We accomplished this with a stochastic figure-ground task (SFG: [36]), in which listeners detect a synthetic auditory object with elements at multiple frequencies in a background of similar noise. The stimulus (Fig. 1) starts with a background of random frequency elements (short tone pips) in frequency-time space; at some point, a number of these elements exhibit a fixed frequency over time (“Figure + Ground Example” in Fig. 1), constituting the object. The listener’s task is to detect whether an object occurred (on half the trials, there is no object, just random frequency elements: “Ground-only Example” in Fig. 1).

In NH listeners, behavioral measures of SFG perception correlated with speech-in-noise ability independently of the audiometric thresholds, which ranged between −10 and 20 dB SPL in the frequency range of 250–8000 Hz, when the SFG stimuli were presented at a fixed level for all the participants [24], validating the crucial role of this ability for speech-in-noise perception. Previous studies support the idea that a possible mechanism that makes the SFG task doable is detecting temporal coherence between figure elements [37], which occurs in and beyond the auditory cortex [36, 38, 39]. Electrical hearing in CI users preserves the temporal envelope of the signal in different frequency bands, while limiting the temporal fine structure cues. In principle, such a mechanism that utilizes temporal coherence of multiple frequency components across different channels could also allow CI users to detect the figure elements in the SFG stimuli. This study aimed to measure individual differences in the ability of CI users to detect figures that are encoded electrically and test the correlation of these with speech-in-noise performance that is not mediated by peripheral fidelity.

Forty-seven post-lingually deafened CI users performed a sentence-in-noise understanding task (AzBio: [40]) along with a SFG detection task. Our experiment had to address two further concerns.

First, our CI users span a range of devices, and many supplement the electrical hearing of the CI with acoustic hearing from an ispi- or contra-lateral hearing aid (hybrid or bimodal listeners, respectively). To control for these differences, and to determine whether such grouping mechanisms are available based on the CI input alone, SFG stimuli were constructed to only span the frequency ranges used for electric (CI) hearing.

Second, as mentioned above, a critical factor in speech-in-noise in CI users is the degree of encoding fidelity in the auditory periphery—this is predicted to relate to both speech perception, speech-in-noise ability, and SFG performance. To thus account for these differences, we also assessed encoding fidelity using a spectral ripple discrimination task (which measures the frequency resolution ability: [41, 42]) and a temporal modulation detection task (which measures fidelity in time) for use as additional predictor variables. For our spectral ripple test, to avoid potential aliasing due to the sparse spectral sampling of CI processors when the ripple density is high [43], we fixed the ripple density and varied the depth. A previous study on normal hearing listeners reported an interrelation between the ripple depth and density thresholds [44].

The peripheral and central measures were used as predictor variables of AzBio performance in a multiple linear regression model. Our principal hypothesis is that central grouping of the electrical signal in CI users explains variance in AzBio performance independently of spectral and temporal resolution.

## Materials and Methods

### Participants

Forty-seven CI users, between 20 and 79 years of age (mean = 60.9 years, SD = 12.1 years; median = 63.3 years; 46.8% female), were recruited from the University of Iowa Cochlear Implant Clinical Research Center. Demographic and audiological characteristics were obtained from clinical records. All the participants were neurologically normal. The average length of device use was 39.5 months (SD = 56.8 months). The average duration of deafness (i.e., patients’ experience of severe hearing loss) was 22.0 years (SD = 15.0 years). Five subjects were bilateral CI users. Among the remaining subjects, 66.1% had a CI in the right ear. Most of the current CI sample had some residual acoustic hearing usually in the low frequency ranges. A minority (23.7%) used bimodal configurations (electric stimulation in one ear and acoustic in the other) while the majority (76.3%) used a hybrid configuration (electric and acoustic stimulation within the same ear). Their hearing aids were in place during testing. The average threshold of low-frequency (i.e., 250 and 500 Hz) residual acoustic hearing in the better ear was 59.4 dB HL (SD = 20.5 dB HL). All CI users had post-lingual onset of deafness (i.e., onset of hearing loss later than 16 years old) and spoke American English as their primary language. See Supplementary Table 1 for the list of participants and their demographic information.

Most participants were tested during the same day as a clinical visit in which they received an annual audiological examination and device tuning. All participants were tested in the best-aided condition, which is the one they use most often in real life. All study procedures were reviewed and approved by the local Institutional Review Board. All the participants provided written informed consent.

### Task Design and Procedures

All CI users performed the spectral ripple discrimination, temporal modulation detection, SFG, and speech in noise (AzBio) tasks. All tasks were performed in a double-walled sound booth using sound-field presentation from a single LOFT40, JBL speaker in the midline placed 1.5 m from the subject.

### Speech-in-Noise: AzBio

Performance on a sentence-in-noise task (AzBio: [40]) was used as a dependent variable in the later multiple linear regression analysis to predict CI individuals’ speech-in-noise ability. Our AzBio task was performed at +5 dB SNR at 70 dB SPL. Subjects heard a sentence and had to repeat it aloud. Outside of the sound booth, an audiologist counted the number of correctly repeated words. Performance was calculated as the ratio of correctly repeated words to the total number of words in all the twenty presented sentences.

### Spectral Ripple and Temporal Modulation

Both the spectral ripple and temporal modulation tasks used an Updated Maximum-Likelihood (UML) adaptive procedure. On each trial, participants performed an oddball task in which they heard three sounds and indicated which differed from the other two in either spectral peak (i.e., the phase of spectral ripple) or modulation frequency (see below). Stimuli for both tasks were generated in MATLAB at the time of testing. The discrimination sequence used an Updated Maximum-Likelihood (UML) adaptive procedure [45]. UML is a Bayesian adaptive procedure which estimates a psychophysical function on each trial and uses the current estimate to identify the stimulus (e.g., the degree of ripple depth) that would be optimally informative to test on the next trial. This can lead to more robust estimates of performance with fewer trials than traditional staircase procedures.

Our implementation assumed a three-parameter logistic as the psychometric function with free parameters for threshold (which captures something akin to the just noticeable difference), slope (sensitivity), and guess rate. The crossover (expressed in terms of dB of depth) was used as our primary estimate of an individual’s perceptual fidelity on each dimension. That is, crossover indicates discrimination ability along spectral and temporal dimensions in each respective task.

Priors (mean and SD) of all three parameters were based on pilot data from 40 CI users. In the UML, the initial stimulus is governed by the priors, and after each response, the psychophysical function is refit. Subsequent trials are then adaptively generated based on the predictions of the UML procedure given the subject’s responses. Unlike traditional tasks, the UML procedure adaptively predicts what to test to best estimate an individual’s psychometric function.

For the *spectral ripple task*, the ripple stimulus was broadband noise that was sinusoidally modulated in log-frequency space. Ripple density was 1.25 ripples per octave—a low density meant to capture the kind of spectral shapes relevant to speech (e.g., the formants of a vowel) and avoid CI-related artifacts at high densities [43]. The amplitude depth of the ripples (in dB) was manipulated based on the UML predictions. On each trial, two standard sounds were created with a randomized starting location for the spectral peak, and the oddball was created with an inverted phase to be maximally distinct. Each trial’s standard and oddball intervals had the same ripple depth.

For the *temporal modulation detection task*, the stimulus was a five-component sound with frequencies at 1515, 2350, 3485, 5045, and 6990 Hz. The whole sound was sinusoidally amplitude modulated at a rate of 20 Hz, and the modulation depth was determined by UML prediction. Trials either had two modulated sounds, where the oddball was unmodulated, or two unmodulated sounds, where the oddball was modulated.

Stimuli for both tasks were 500 ms in duration and linearly ramped with a 50 ms rise/fall. To compensate for intensity differences in the modulated stimuli, root mean square values were equalized, and the presentation level was roved randomly across the three sounds by between −3 and +3 dB. This randomness should deter the use of loudness as a reliable cue.

The task was a 3-interval, 3-alternative forced-choice oddball detection paradigm. The task was implemented using Psychtoolbox 3 [46] in MATLAB (The Mathworks). On each trial, two standard stimuli and one oddball were played in random order with an ISI of 750 ms. A numbered box appeared on the computer screen as each stimulus played. Subjects were instructed to choose the token that differed from the other two. Responses could be made by numeric keypad or by mouse-click within the corresponding box on the screen. The UML approach allowed the tasks to be much shorter than traditional staircase measures; each task was 70 trials. Both tasks began with 4 practice trials to familiarize the subject with the procedure, and correct/incorrect feedback was given on every trial.

### SFG

The *SFG stimuli* were generated as in [37]. Each time-segment contained a fixed number of components at random frequencies in log-frequency space. In trials containing a figure, a proportion of the components were constrained to remain the same over each time segment to create a figure with fixed frequency components that subjects were required to detect among a random background of frequency components. All the tone pips were constrained to be above 1 kHz so that even for subjects with residual low-frequency hearing, figure detection required only the electric range (and the acoustic hearing would most likely be unhelpful). The stimulus therefore assessed electrical grouping in all subjects, regardless of their hearing configuration. The spectral separation of elements was constrained to be at least a half octave to reduce the likelihood of frequency resolution abilities confounding the results. Figure 1A shows example spectrograms of ground-only and figure + ground stimuli. Figure 1B shows the electrodograms of example SFG stimuli, generated based on the 22-channel Cochlea device with the ACE sound coding strategy. Section 2.2 of Yang et al. [17] describes how the electrodograms are generated. Using the electrodograms, we compared integrated current levels between all the Ground-only and Figure+Ground stimuli in the 2–4 s period (where the emergence of a “figure” is expected). No significance difference was found between the current levels (Mann-Whitney Rank Sum test, *p* = 0.94), indicating that the overall current level difference could not be used to perform the task (Fig. 1C).

All stimuli were created using MATLAB software (The Mathworks) at a sampling rate of 44.1 kHz and 16-bit resolution. Extensive piloting with CI listeners was conducted to determine stimulus characteristics that were never associated with floor or ceiling effects. We used a stimulus that consisted of 50-ms segments, each containing eight frequency components. The whole stimulus was 4 s-long. For the first half (ground portion) of 40 segments, each segment was created from a selection of eight separate randomly selected frequencies drawn from a distribution of 145 components separated by 1/48th of an octave across 1–8 kHz. On a “ground” trial, the second half comprised 40 segments constructed in the same way as the first half. On a “figure” trial (see Fig. 1), the second half of 40 segments was constructed from components in which six of the eight stayed at the same frequency to create a “figure”. The other two components were selected at random frequencies.

**Fig. 2:**
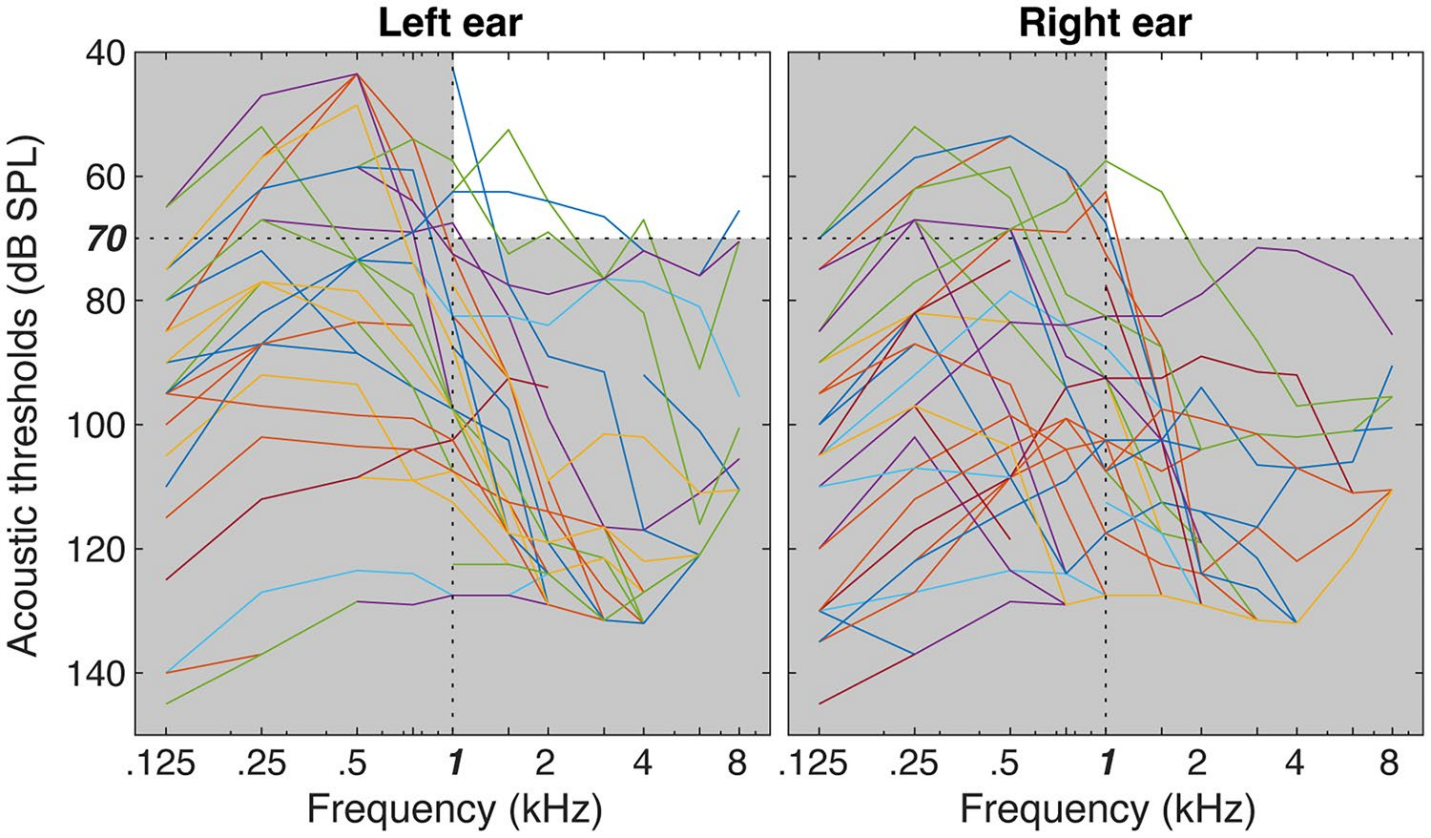
Residual acoustic hearing thresholds of all the participants represented in dB *SPL* as a function of stimulus frequency. The hearing thresholds were measured without a CI or hearing aids. The white areas depict the audibility zone of our SFG stimuli (i.e., above 70 dB SPL, above 1 kHz)

The *SFG task* was implemented in custom-written MATLAB scripts (The Mathworks) using Psychtoolbox 3 [46]. Instructions were presented via a computer monitor located 0.5 m in front of the subject at eye level. Sound levels were the same across subjects, presented at 70 dB SPL. At this presentation level, very few participants could use their residual acoustic hearing to hear the SFG stimuli; see the white areas in Fig. 2 that depicts the audibility zone of our SFG stimuli (i.e., above 70 dB SPL, above 1 kHz).

On each trial, participants saw the trial number displayed for 600 ms. This then cleared to display a fixation cross for 1 s before the start of the sound. After the sound and a 100 ms pause, a text prompt to respond was shown on the screen (‘Target? 1: Yes, 2: No’). Subjects then had up to 10 s to respond by a numeric keypad to indicate if a figure was detected. Once a response was recorded, the fixation cross was shown, and a delay of 600 ms occurred until the start of the next sound. One hundred and twenty trials were presented with a figure occurring in a random half; a break was given after 40 trials. One hundred and twenty unique different stimuli were pre-generated and presented in a random order. All the subjects were presented with the same set of 120 stimuli but in a different order.

### Statistical Analyses

Initial exploratory analyses related each predictor to each other and to AzBio performance using bivariate correlations. Our primary analysis related each predictor to speech perception performance on AzBio using multiple regression to assess the impact of SFG while controlling for the periphery. The final model is given in (1), in the syntax of the regression function in R (lm()).

1 $$Speech\; Perception \sim 1+SFG+{SpecRipple}+TempMod$$

Here, Speech Perception is accuracy on the AzBio task, SFG is performance on the SFG task expressed in terms of d’. SpecRipple and TempMod refer to the crossover parameter of the psychophysical discrimination function expressed in dB of depth.

## Results

### Evaluation of Independent Variables in Bivariate Analyses

**Fig. 3:**
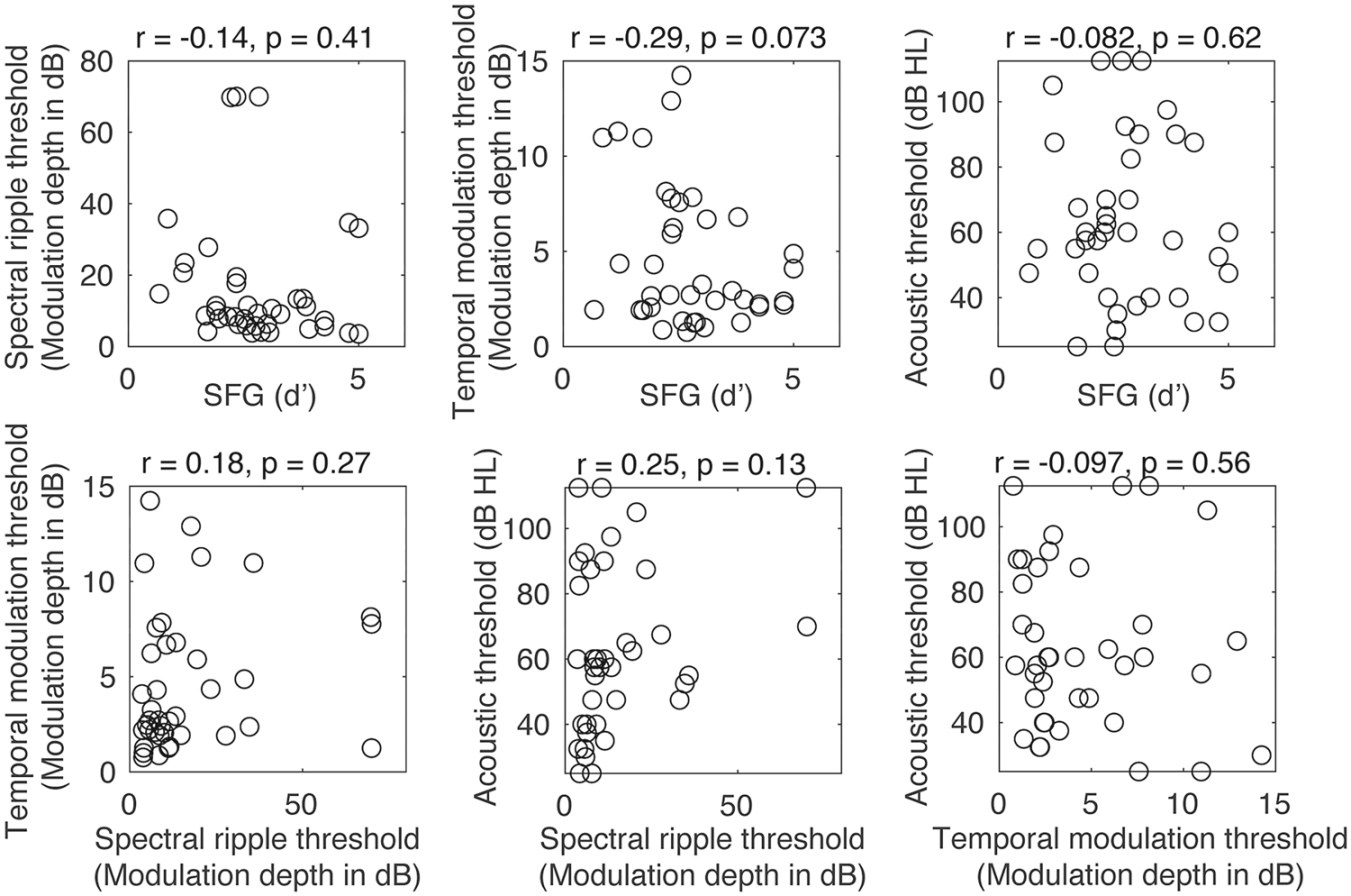
Results from predictor co-linearity analysis. Acoustic threshold: better-ear low-frequency (250 and 500 Hz) residual acoustic hearing thresholds. Temporal and spectral modulation thresholds are expressed in dB (depth of the modulation). No significant correlations were observed

We started by evaluating the correlations among all the independent variables to check for co-linearity prior to multiple linear regression analysis. No significant correlations were found between any predictor variables. The relationship between the predictor variables is shown in Fig. 3 as scatter plots. This showed first that spectral and temporal fidelity were uncorrelated, suggesting (as predicted) that they comprise two independent dimensions of auditory encoding fidelity in CI users. Second, SFG performance was not correlated with spectral fidelity and only trending toward a significant correlation with temporal fidelity. This suggests that—also as expected—the stimuli that were used did not strongly relate to peripheral fidelity for CI users. In addition, we compared the average threshold of low-frequency (i.e., 250 and 500 Hz) residual acoustic hearing in the better ear to the predictor variables, as shown in the bottom panels of Fig. 3. No correlation was found between the residual acoustic hearing thresholds and the independent variables.

**Fig. 4:**
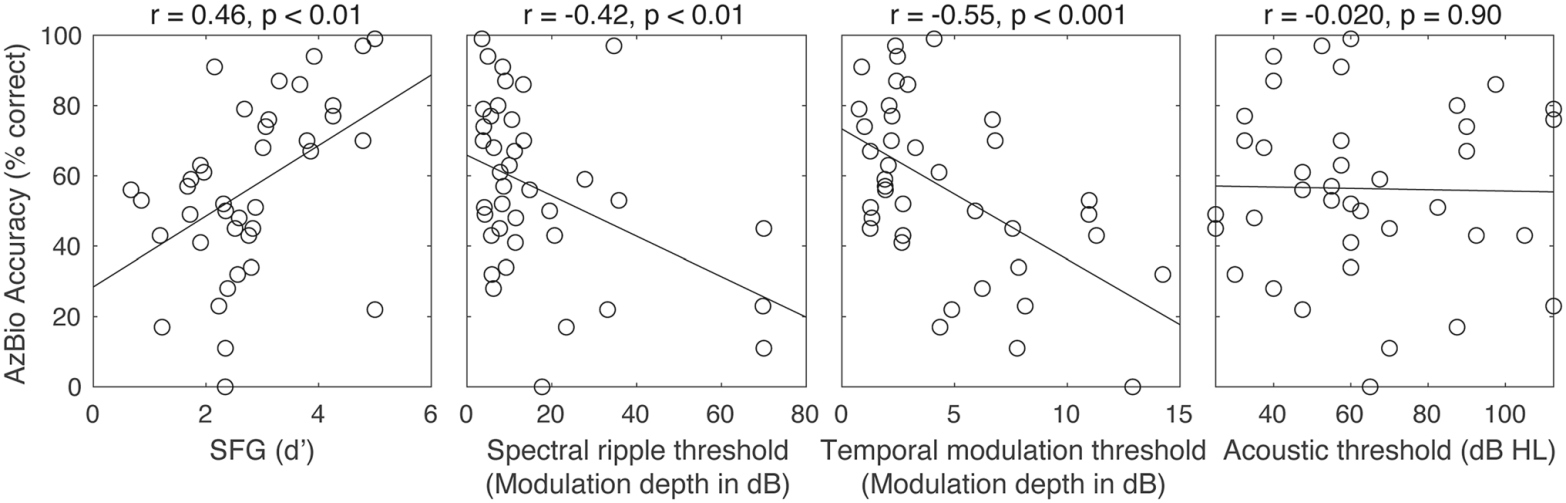
Results from bivariate correlation analyses. Acoustic threshold: better-ear low-frequency (250 and 500 Hz) residual acoustic hearing thresholds

To test the next assumption for multiple regression that the independent variables should be correlated with the dependent variable, we conducted bivariate analyses examining correlations between each independent variable and AzBio accuracy. SFG, spectral, and temporal fidelity exhibited a statistically significant correlation with speech-in-noise ability. However, residual acoustic hearing thresholds did not; thus, we did not use the acoustic thresholds as a predictor variable in the following multiple linear regression analysis. These are shown in Fig. 4. In all three cases with significant correlations, better performance (higher SFG, lower ripple or temporal modulation threshold) predicted better AzBio performance.

### Multiple Linear Regression

**Fig. 5:**
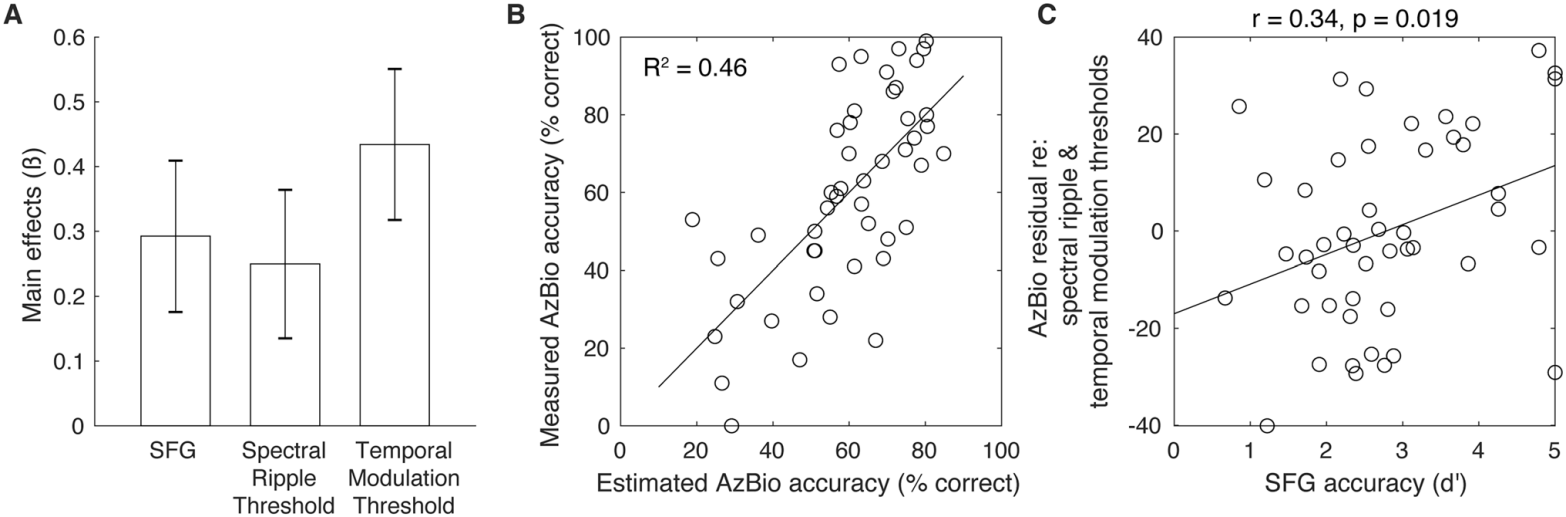
Results from multiple linear regression analysis. **A** Main effects of predictor variables. **B** Relationship between estimated AzBio accuracy (i.e., the model output) and measured AzBio accuracy (i.e., the dependent variable). **C** Relationship between SFG accuracy and the residual of AzBio accuracy after regressing out the other two predictor variables (i.e., spectral and temporal resolution)

**Fig. 6:**
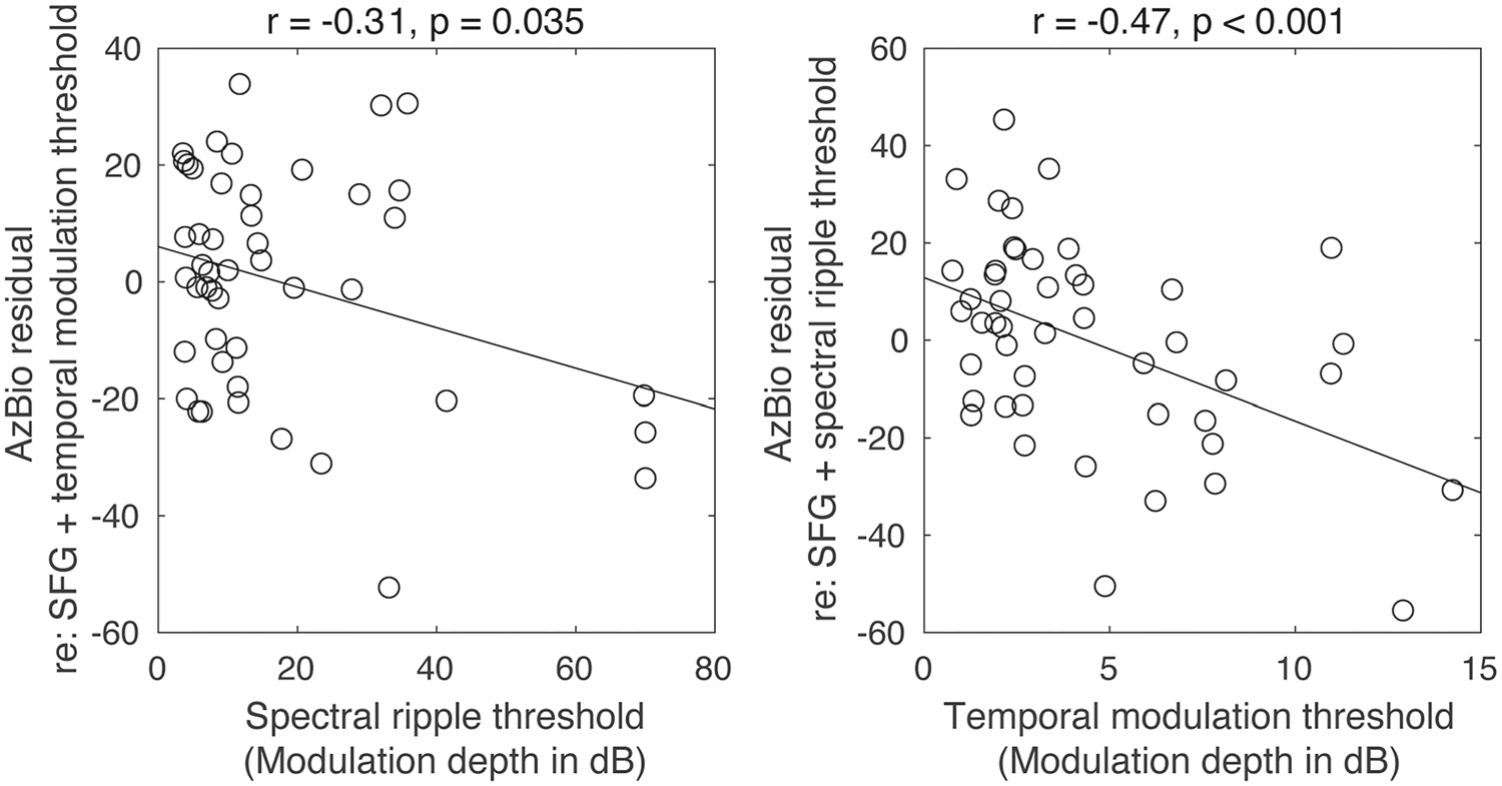
Relationship of spectral ripple and temporal modulation thresholds with the residual of AzBio accuracy after regressing out the other two predictor variables

Following bivariate analyses, we conducted a multiple linear regression analysis to determine which of the independent variables predicted AzBio accuracy when accounting for all others (see Table 1 and Fig. 5A). When adjusted for the number of independent variables, the model accounted for 46.3% of the variance in AzBio accuracy (see Fig. 5B), *F*(3, 43) = 12.4, *p* < 0.00001, adjusted *R*^2^ = 0.426. All three predictors reached statistical significance. Critically, the effect of SFG was significant—and positively related to outcomes—even after accounting for the auditory periphery (Fig. 5C). This was the same for the spectral ripple and the temporal modulation thresholds; as shown in Fig. 6, each predictor variable showed a significant correlation even after regressing out the other predictor variables.

**Table 1:** Results from multiple linear regression on speech-in-noise accuracy (*N* = 47, *R*^2^ = 0.463)

AzBio	*β* (normalized)	SE	T(43)	*p*	Partial *ρ*
SFG	0.292	0.117	2.51	0.0160	0.357
Spectral ripple thresholds	−0.250	0.114	−2.18	0.0345	−0.316
Temporal modulation thresholds	−0.434	0.117	−3.72	< 0.001	−0.493

## Discussion

In this study, post-lingually deafened CI users performed a SFG task in which listeners detected temporally coherent frequency components against a random background. The bivariate correlation between figure-detection performance (d-prime) and sentence-in-noise performance (AzBio score) reached *r* = 0.45 (*p* < 0.005). Moreover, multiple linear regression demonstrated a significant effect of figure detection (normalized beta coefficient = 0.29, *p* < 0.05) even after accounting for the fidelity of spectral and temporal encoding in the auditory periphery. The combined model explained 46% of the variance in speech-in-noise performance. This work has therefore established a relationship between a simple measure of the cross-frequency grouping of electrically coded signals to speech-in-noise ability.

This result suggests an auditory-cognitive mechanism of auditory grouping as one of the factors that contributes to speech-in-noise performance. Adopting the SFG task in clinics may reveal a source of speech-in-noise difficulty in CI users. For example, the SFG stimuli can be adjusted to make the figure elements occur in the specific frequency range to be tested, or occur across two different devices (e.g., electric and acoustic) so that the perceptual fusion across devices can be tested. When combined with device reprogramming or perceptual training, the SFG task may test the change in the cross-electrode processing. It is also advantageous that the SFG task is language independent, although it means that language-specific abilities would be un-tested by this task.

The relatively large sample size in this study provided an opportunity to investigate the relative contributions of spectral and temporal resolution to the prediction of speech-in-noise performance through multiple linear regression. The correlation between speech-in-noise performance and spectral [14] and temporal resolution [26, 47, 48] has been reproduced well by this study, although it should be noted that most previous studies that reported a relationship between spectral resolution and speech perception varied the spectral ripple *density*, not depth. In this study, temporal resolution showed stronger correlation with speech-in-noise performance than spectral resolution, as well as greater contribution to the prediction of speech-in-noise in the linear regression model. This finding is consistent with many previous studies that showed the importance of temporal envelope encoding in CIs for successful speech perception [48–51]. However, this finding (i.e., temporal resolution demonstrating greater importance than spectral) is inconsistent with a previous study that directly compared the correlations of spectral and temporal resolution with speech-in-noise performance and showed a better correlation of spectral resolution (e.g., [26]). This inconsistency can be due to the difference in the spectral resolution test (i.e., varying ripple density vs. varying depth), the difference in the stimuli (AzBio sentences in this study vs. single words in [26]) or CI device types.

The figure detection ability during the SFG task is unlikely the only auditory cognitive mechanism that contributes to speech-in-noise performance. Although forty-seven is a relatively large sample size for a CI study, the number of predictor variables was limited to three to ensure reasonable statistical power. A future larger study should consider more auditory-cognitive mechanisms (e.g., auditory working memory: [52–54], auditory selective attention: [33, 34, 55]) as well as linguistic and general cognitive mechanisms [56].

We carefully designed the stimuli for the SFG task so that they are only perceived in the electric hearing region. This was to control the different level of residual acoustic hearing among subjects. A future study may focus on the contribution of the residual or contralateral acoustic hearing, also its integration with electric hearing, to the figure detection during the SFG task.

This study has a few limitations. First, it is possible that the SFG ability captured different kinds of auditory periphery fidelity that were not reflected in spectral ripple and temporal modulation discrimination tasks. For example, the electrode-neuron interface could be poorer in some CI users than others [57–59], which could result in degraded SFG and speech-in-noise perception. To rule out this alternative interpretation, a future study should utilize an electrophysiological measure of peripheral encoding.

Second, although we carefully engineered the frequency range, level, and the frequency distance between the elements of our SFG stimuli, “equal electrical hearing” is still not guaranteed due to the heterogeneity of device types. For example, loudness summation between electrodes can be different for different CI devices. To avoid this confounding factor, a future study may (1) test a cohort of the same device type or (2) utilize electrodograms to quantify the device differences and use the measure as a predictor variable. It has to be noted that the electrodogram in Fig. 1B is for a representative device. It does not account for the differences in the device types and the variance of electrode-neuron interface. Some individuals whose electrodes are not perfectly matched for level could use loudness cues. Also, any hearing aids could be turned off during the SFG task to further prevent the contribution of acoustic hearing.

Our future studies will follow our CI participants to examine changes in their SFG ability along with the changes in their peripheral encoding acuity (as in previous studies that have monitored changes in CI peripheral encoding over time: [60, 61]) as well as speech-in-noise performance. This longitudinal study will help us dissociate the contributions of the periphery to SFG ability, if their pattern of change differs over time. Also, a future study can use the SFG task for auditory perceptual training after cochlear implantation. For example, the auditory “figure” can be presented with simultaneous visual cues until the auditory system *learns* how to detect the figure.

### Supplementary Information

Below is the link to the electronic supplementary material.

Supplementary file1 (XLSX 56 KB)
**Supplementary Table 1** List of subjects

## Data Availability

Data will be shared upon requests.

## References

[1] Gantz BJ, Dunn C, Oleson J, Hansen M, Parkinson A, Turner C (2016). Multicenter clinical trial of the Nucleus Hybrid S8 cochlear implant: final outcomes. Laryngoscope.

[2] Finley CC (2008). Role of electrode placement as a contributor to variability in cochlear implant outcomes. Otol Neurotol.

[3] Seyyedi M, Nadol JB (2014). Intracochlear inflammatory response to cochlear implant electrodes in the human. Otol Neurotol.

[4] Goehring T, Archer-Boyd A, Deeks JM, Arenberg JG, Carlyon RP (2019). A site-selection strategy based on polarity sensitivity for cochlear implants: effects on spectro-temporal resolution and speech perception. J Assoc Res Otolaryngol.

[5] Bierer JA, Faulkner KF (2010). Identifying cochlear implant channels with poor electrode-neuron interface: partial tripolar, single-channel thresholds and psychophysical tuning curves NIH Public Access. Ear Hear.

[6] Bierer JA, Litvak L (2016). Reducing channel interaction through cochlear implant programming may improve speech perception: current focusing and channel deactivation. Trends Hear.

[7] Vickers D, Degun A, Canas A, Stainsby T, Vanpoucke F (2016) Deactivating cochlear implant electrodes based on pitch information for users of the ACE strategy. In: van Dijk P, Başkent D, Gaudrain E, de Kleine E, Wagner A, Lanting C (eds) Physiology, psychoacoustics and cognition in normal and impaired hearing. Advances in experimental medicine and biology, vol 894. Springer, Cham., pp 115–123. Available from http://www.springer.com/series/5584 10.1007/978-3-319-25474-6_13 27080652

[8] Dawson PW, McKay CM, Busby PA, Grayden DB, Clark GM (2000) Electrode discrimination and speech perception in young children using cochlear implants. Ear Hear 21(6):597–607. Available from http://journals.lww.com/ear-hearing 10.1097/00003446-200012000-00007 11132786

[9] Jeon EK, Turner CW, Karsten SA, Henry BA, Gantz BJ (2015). Cochlear implant users’ spectral ripple resolution. J Acoust Soc Am.

[10] Davies-Venn E, Nelson P, Souza P (2015). Comparing auditory filter bandwidths, spectral ripple modulation detection, spectral ripple discrimination, and speech recognition: Normal and impaired hearing. J Acoust Soc Am.

[11] Landsberger DM, Padilla M, Martinez AS, Eisenberg LS (2018). Spectral-temporal modulated ripple discrimination by children with cochlear implants. Ear Hear.

[12] Jones GL, Ho Won J, Drennan WR, Rubinstein JT (2013). Relationship between channel interaction and spectral-ripple discrimination in cochlear implant users. J Acoust Soc Am.

[13] Anderson ES, Nelson DA, Kreft H, Nelson PB, Oxenham AJ (2011). Comparing spatial tuning curves, spectral ripple resolution, and speech perception in cochlear implant users. J Acoust Soc Am.

[14] Won JH, Drennan WR, Rubinstein JT (2007). Spectral-ripple resolution correlates with speech reception in noise in cochlear implant users. J Assoc Res Otolaryngol.

[15] Litvak LM, Spahr AJ, Saoji AA, Fridman GY (2007). Relationship between perception of spectral ripple and speech recognition in cochlear implant and vocoder listeners. J Acoust Soc Am.

[16] Bingabr M, Espinoza-Varas B, Loizou PC (2008). Simulating the effect of spread of excitation in cochlear implants. Hear Res.

[17] Yang H, Won JH, Choi I, Woo J (2020). A computational study to model the effect of electrode-to-auditory nerve fiber distance on spectral resolution in cochlear implant. PLoS ONE.

[18] Bierer JA, Deeks JM, Billig AJ, Carlyon RP (2015). Comparison of signal and gap-detection thresholds for focused and broad cochlear implant electrode configurations. J Assoc Res Otolaryngol.

[19] Hamzavi J, Baumgartner WD, Pok SM, Franz P, Gstoettner W (2003). Variables affecting speech perception in postlingually deaf adults following cochlear implantation. Acta Otolaryngol.

[20] Fetterman BL, Domico EH (2002). Speech recognition in background noise of cochlear implant patients. Otolaryngol Head Neck Surg.

[21] Noble W, Tyler RS, Dunn CC, Bhullar N (2009). Younger- and older-age adults with unilateral and bilateral cochlear implants: speech and spatial hearing self-ratings and performance. Otol Neurotol.

[22] Bregman AS (1994). Auditory scene analysis: the perceptual organization of sound.

[23] Ruggles D, Bharadwaj H, Shinn-Cunningham BG (2011). Normal hearing is not enough to guarantee robust encoding of suprathreshold features important in everyday communication. Proc Natl Acad Sci USA.

[24] Holmes E, Griffiths TD (2019). ‘Normal’ hearing thresholds and fundamental auditory grouping processes predict difficulties with speech-in-noise perception. Sci Rep.

[25] Holmes E, Zeidman P, Friston KJ, Griffiths TD (2021). Difficulties with speech-in-noise perception related to fundamental grouping processes in auditory cortex. Cereb Cortex.

[26] Winn MB, Won JH, Moon IJ (2016). Assessment of spectral and temporal resolution in cochlear implant users using psychoacoustic discrimination and speech cue categorization. Ear Hear.

[27] Chatterjee M, Sarampalis A, Oba SI (2006). Auditory stream segregation with cochlear implants: a preliminary report. Hear Res.

[28] Cooper HR, Roberts B (2007). Auditory stream segregation of tone sequences in cochlear implant listeners. Hear Res.

[29] Duran SI, Collins LM, Throckmorton CS (2012). Stream segregation on a single electrode as a function of pulse rate in cochlear implant listeners. J Acoust Soc Am.

[30] Marozeau J, Innes-Brown H, Blamey PJ (2013). The acoustic and perceptual cues affecting melody segregation for listeners with a cochlear implant. Front Psychol.

[31] Paredes-Gallardo A, Madsen SMK, Dau T, Marozeau J (2018). The role of temporal cues in voluntary stream segregation for cochlear implant users. Trends Hear.

[32] Paredes-Gallardo A, Madsen SMK, Dau T, Marozeau J (2018). The role of place cues in voluntary stream segregation for cochlear implant users. Trends Hear.

[33] Nogueira W, Dolhopiatenko H (2022). Predicting speech intelligibility from a selective attention decoding paradigm in cochlear implant users. J Neural Eng.

[34] Lee JH, Shim H, Gantz B, Choi I (2022) Strength of attentional modulation on cortical auditory evoked responses correlates with speech-in-noise performance in bimodal cochlear implant users. Trends Hear 26:23312165221141144. https://journals.sagepub.com/doi/10.1177/23312165221141143 10.1177/23312165221141143 PMC9726851 36464791

[35] Hong RS, Turner CW (2006). Pure-tone auditory stream segregation and speech perception in noise in cochlear implant recipients. J Acoust Soc Am.

[36] Teki S, Chait M, Kumar S, von Kriegstein K, Griffiths TD (2011). Brain bases for auditory stimulus-driven figure-ground segregation. J Neurosci.

[37] Teki S, Chait M, Kumar S, Shamma S, Griffiths TD (2013). Segregation of complex acoustic scenes based on temporal coherence. Elife.

[38] O’Sullivan JA, Shamma SA, Lalor EC (2015). Evidence for neural computations of temporal coherence in an auditory scene and their enhancement during active listening. J Neurosci.

[39] Teki S, Barascud N, Picard S, Payne C, Griffiths TD, Chait M (2016). Neural correlates of auditory figure-ground segregation based on temporal coherence. Cereb Cortex.

[40] Spahr AJ (2012). Development and validation of the AzBio sentence lists. Ear Hear.

[41] Archer-Boyd AW, Southwell RV, Deeks JM, Turner RE, Carlyon RP (2018). Development and validation of a spectro-temporal processing test for cochlear-implant listeners. J Acoust Soc Am.

[42] Aronoff JM, Landsberger DM (2013). The development of a modified spectral ripple test. J Acoust Soc Am.

[43] Winn MB, O’Brien G (2022). Distortion of spectral ripples through cochlear implants has major implications for interpreting performance scores. Ear Hear.

[44] Supin AY, Popov VV, Milekhina ON, Tarakanov MB (1999). Ripple depth and density resolution of rippled noise. J Acoust Soc Am.

[45] Shen Y, Dai W, Richards VM (2015). A MATLAB toolbox for the efficient estimation of the psychometric function using the updated maximum-likelihood adaptive procedure. Behav Res Methods.

[46] Brainard DH (1997). The psychophysics toolbox. Spat Vis.

[47] Shannon RV (1992). Temporal modulation transfer functions in patients with cochlear implants. J Acoust Soc Am.

[48] Won JH, Drennan WR, Nie K, Jameyson EM, Rubinstein JT (2011). Acoustic temporal modulation detection and speech perception in cochlear implant listeners. J Acoust Soc Am.

[49] Nie K, Barco A, Zeng FG (2006). Spectral and temporal cues in cochlear implant speech perception. Ear Hear.

[50] Won JH (2012). The ability of cochlear implant users to use temporal envelope cues recovered from speech frequency modulation. J Acoust Soc Am.

[51] Luo X, Fu QJ, Wei CG, Cao KL (2008). Speech recognition and temporal amplitude modulation processing by Mandarin-speaking cochlear implant users. Ear Hear.

[52] Akeroyd MA (2008). Are individual differences in speech reception related to individual differences in cognitive ability? A survey of twenty experimental studies with normal and hearing-impaired adults. Int J Audiol.

[53] Dryden A, Allen HA, Henshaw H, Heinrich A (2017) The association between cognitive performance and speech-in-noise perception for adult listeners: a systematic literature review and meta-analysis. Trends Hear 21:2331216517744675. https://journals.sagepub.com/doi/10.1177/2331216517744675 10.1177/2331216517744675 PMC5734454 29237334

[54] Kim S, Choi I, Schwalje AT, Kim K, Lee JH (2020). Auditory working memory explains variance in speech recognition in older listeners under adverse listening conditions. Clin Interv Aging.

[55] Paul BT, Uzelac M, Chan E, Dimitrijevic A (2020). Poor early cortical differentiation of speech predicts perceptual difficulties of severely hearing-impaired listeners in multi-talker environments. Sci Rep.

[56] Drennan WR, Won JH, Timme AO, Rubinstein JT (2016). Non-linguistic outcome measures in adult cochlear implant users over the first year of implantation. Ear Hear.

[57] Pfingst BE (2015). Importance of cochlear health for implant function. Hear Res.

[58] He S, Skidmore J, Koch B, Chatterjee M, Carter BL, Yuan Y (2023). Relationships between the auditory nerve sensitivity to amplitude modulation, perceptual amplitude modulation rate discrimination sensitivity, and speech perception performance in postlingually deafened adult cochlear implant users. Ear Hear.

[59] Gransier R, Luke R, Van Wieringen A, Wouters J (2020). Neural modulation transmission is a marker for speech perception in noise in cochlear implant users. Ear Hear.

[60] Fayed EA, Saad Zaghloul H, Morgan AE (2020). Electrode impedance changes over time in MED El cochlear implant children recipients: relation to stimulation levels and behavioral measures. Cochlear Implants Int.

[61] Mathew R (2018). Development of electrophysiological and behavioural measures of electrode discrimination in adult cochlear implant users. Hear Res.

